# Tacrolimus ointment in the treatment of hormone-dependent dermatitis: A protocol of systematic review

**DOI:** 10.1097/MD.0000000000022159

**Published:** 2020-09-11

**Authors:** Mao Li, Wen Tan, Jingjing Du, Qiuyue Wang, Linyue Wang, Min Lei, Ping-Sheng Hao

**Affiliations:** a Hospital of Chengdu University of Traditional Chinese Medicine, Chengdu; b Guanghan People's Hospital of Sichuan Province, Deyang, China.

**Keywords:** tacrolimus ointment, facial hormone-dependent dermatitis, RCTs, protocol

## Abstract

**Background:**

Long-term use of corticosteroid ointment for external using or skin management products and cosmetics containing corticosteroid will produce a hormone-dependent effect on facial skin and destroy the barrier function of the skin. It is easy to cause repeated attacks of facial skin inflammation after drug withdrawal because corticosteroid hormones can cause the expression of inflammatory factors in the body, which has a serious impact on patients. The general treatment method is to stop using hormone drugs for psychotherapy and inform patients of the basic knowledge of hormone-dependent dermatitis and daily facial care, but the effect is not good. At present, non-steroidal ointment tacrolimus (a calcineurin inhibitor) is widely used in the treatment of hormone-dependent dermatitis. Tacrolimus ointment is effective for corticosteroid-dependent dermatitis, but adverse events can also occur.

**Methods:**

We plan to searched all randomized controlled trials (RCTs) fortacrolimus ointment therapy of hormone-dependent dermatitis in: MEDLINE, PubMed, EMBASE, the Cochrane Central Register of Controlled Trials (CENTRAL), Springer and Web of Science, China Biomedical Literature Database (CBM), China Science Journal Database (VIP database) and Wanfang Database, China National Knowledge Infrastructure (CNKI), without the limitation of publication status and language until September 1, 2020. The systematic review will also search will also search for identify publications, meeting minutes, and grey literature (including unpublished meeting articles).

**Discussion:**

The systematic review mainly to access the safety and efficacy of tacrolimus ointment for hormone-dependent dermatitis (facial corticosteroid addiction dermatitis and facial steroid dermatitis). The results of our research will facilitate evidence-based management of patients with facial corticosteroid-dependent dermatitis and provide clinical advice on their treatment options.

**Registration:**

PROSPERO CRD42020171813.

## 1 Introduction

Facial hormone dependence dermatitis (HDD) refers to chronic inflammatory lesions on the skin caused by long-term application of corticosteroid-containing drugs and cosmetics.^[1]^ HDD occurs on the thin and tender skin of the face, and clinical symptoms such as burning sensation, tingling, desquamation, erythema, pimples, and blister are mainly seen. Facial HDD directly affects the patients external appearance. In severe cases, it even attracts the attention and criticism of the surrounding people, which causes a severe psychological and physiological burden on the patient. With the extensive use of corticosteroids, the incidence of HDD has increased to 0. 25% in recent years.^[2]^ It can occur at any age, especially in young and middle-aged women, which is closely related to the long-term use of hormone-containing skin management products and cosmetics. The disease has the characteristics of polymorphic damage, hormone dependence or addiction, disease relapse, etc. Improper use or abuse of topical hormones for a long time makes the skin of the affected area dependent on the drug. Once withdrawal, different levels of “withdrawal symptoms” will appear, such as skin flushing, swelling, papules, conscious burning and tension, tingling, and itching, which will increase when exposed to heat or sunlight. According to different clinical manifestations, it can be divided into dermatitis type, acne-like type, pigment type, and aging type.^[3]^ Dermatitis type: skin flushing and thinning, accompanied by telangiectasia; Acne-like type: acne, papule, and pustule; Pigmented type: mainly manifested as pigmentation; Aging type: the performance is dry skin, desquamation, roughness, and even atrophy.^[4–6]^ The pathogenesis of HDD is complex. The pathogenes of HDD is complex. A great many studies^[7–9]^ have shown that its occurrence is mainly related to skin barrier damage, increased inflammatory response, neurological hyper-responsiveness, and microbial infection. Impaired skin barrier function is the main reason for the hard disk drive. It includes the following aspects: topical corticosteroid ointment causes dermal small blood vessel dysfunction, causing capillary dilation; long-term local use of hormones inhibits the migration and proliferation of epidermal cells, resulting in keratin Layer thinning, skin atrophy, and reduced skin barrier function; the immunosuppressive effect of corticosteroids weakens the local immune function of the skin, increases the sensitivity of the skin, and increases the risk of microbial and bacterial infections. At present, there is no exact and effective treatment for this disease. The point of treatment is to eliminate the dependence of patients on corticosteroids. In the early stage, corticosteroid decreasing or replacement therapy was used; topical use of skin barrier repair milk, non-steroidal drugs, calcium-regulated nerve phosphates inhibitors, etc., of which tacrolimus ointment is the most common; at the same time, antihistamines and immunomodulators were used to inhibit the inflammatory reaction and reduce neurovascular hyper-responsiveness; also, it can also cooperate with intense pulsed light therapy to repair skin barrier function. The treatment of this disease is more difficult because of its stubbornness, long treatment period, easy relapse after drug withdrawal, and high incidence of adverse reactions.

Tacrolimus ointment, a topically applied macrolide lactone immunomodulator, is for facial steroid dermatitis.^[10]^ Dene Simpson et al^[11]^ believe that “ tacrolimus ointment is well tolerated by people with atopic dermatitis, especially when long-term treatment is needed or wrinkled areas of the face or skin are involved.” Studies^[11–14]^ have shown tacrolimus ointment is also for psoriasis vulgaris, lichenplanus, rosacea, contact dermatitis, seborrhoeic dermatitis, land vitiligo.

The immunomodulatory and anti-inflammatory Pharmacological Propertiesry properties of Tacrolimus ointment has demonstrated in animal models and human studies.^[11]^ Tacrolimus ointment has the characteristics of good skin permeability and small molecular weight, which mechanism of action mainly involves calcineurin inhibition. Experimental evidence suggests that one of the ways that tacrolimus inhibits the activation of T-lymphocytes is to bind to the intracellular protein FKBP-12.^[12–15]^ The release of factors and inflammatory mediators produces anti-inflammatory effects. At the same time, through the release of skin mast cells and mediators with basophilic granulocytes, it effectively promotes collagen synthesis of the skin. It restores the original barrier function of the skin.

Topical corticosteroid is widely used in clinical practice, which is a milestone in dermatology external drug treatment. It has the characteristics of quick action and strong anti-inflammatory effects, while the side effects are often ignored by people. At present, there are more and more cases of recurrence and aggravation of skin diseases caused by the sudden withdrawal of corticosteroid-containing preparations after long-term external use. Clinicians often use tacrolimus, a calcineurin inhibitor, to relieve facial itching and redness of patients. A majority of clinical studies have suggested that tacrolimus ointment is effective for hormone-dependent dermatitis, but there is no systematic evaluation of tacrolimus for corticosteroid-dependent dermatitis. It is reported that skin burning or itching may occur after using tacrolimus in clinical application, and the incidence of itching in children using tacrolimus is similar to that of children using 1% hydrocortisone acetate ointment. In adults, tacrolimus ointment is more prone to itching than 0.1% hydrocortisone butyrate ointment.^[16]^ Therefore, using more rigorous search strategies, more objective results evaluation, and strict review methods, we hope that our systematic review will provide more convincing conclusions.

## 2 Methods

The protocol of this systematic review has been registered in the PROSPERO website (CRD42020171813).

### 2.1 Inclusion criteria

#### 2.1.1 Types of studies

The randomized controlled trials (RCTs) without the limitations of language and publicity will be included. However, quasi-randomized trials and random crossover studies will be excluded.

#### 2.1.2 Types of participants

According to the following criteria, patients with facial hormone-dependent dermatitis are not limited by age, race, sex, or educational and economic status:

Inclusion criteria:

1. A month or more application history of corticosteroid-containing cosmetics corticosteroid ointment and skin management products on the face; the clinical symptoms are tingling, itching, burning, and other symptoms.
2. Facial skin lesions are dependent on hormonal cosmetics, etc. The original skin lesions can be aggravated after being discontinued for 3 to 5 days and can be relieved after continued application.
3. Meet the above diagnosis.
4. Patients who voluntarily entered the group and signed informed consent after understanding the content of the study.

Exclusion criteria:

1. Patients who with facial seborrheic dermatitis, acne vulgaris, and other facial skin diseases;
2. Patients who with facial skin dysfunction;
3. Patients who with combined immune dysfunction and long-term application of hormone drugs;
4. Patients who with severe diseases or insufficiency of heart, brain, lung, liver, kidney, and other organs, patients with original hypertension;
5. Patients who with acute stage of infectious disease;
6. Patients who with trauma and surgical recovery;
7. Patients who with physiological time during pregnancy and lactation;
8. Patients who with mental illness.

#### 2.1.3 Types of interventions

The intervention group was mainly treated with tacrolimus ointment.

1. Tacrolimus ointment treatment compared with no treatment.
2. Tacrolimus ointment therapy compared to placebo therapy.
3. Tacrolimus ointment therapy compared to other ointment therapies.

### 2.2 Types of outcome measures

#### 2.2.1 Primary outcomes

The main result is efficiency. According to the severity of clinical symptoms, it is divided into 4 quartiles, and the scores are the sum of the scores. Symptom reduction index (SSRI) = (score before treatment-score after treatment)/score before treatment × 100%. After SSRI is cured, the clinical symptoms are reduced by >95%, the significant effect is from 70% to 95%, the effective is from 30% to <70%, and the ineffectiveness is from <30%; the number of excluded cases is completely effective.

#### 2.2.2 Additional outcomes

1. Indicators of skin barrier function, including cuticle moisture content, epidermal moisture loss, and sebum content.
2. Serum indicator test.
3. Adverse events of the treatment.
4. Hormone-dependent dermatitis relapse rate.

#### 2.2.3 Search methods for identification of studies

We plan to searched all randomized controlled trials (RCTs) fortacrolimus ointment therapy of hormone-dependent dermatitis in: MEDLINE, PubMed, the Cochrane Central Register of Controlled Trials (CENTRAL), Web of Science, Springer, and EMBASE, China Biomedical Literature Database (CBM), China Science Journal Database (VIP database) and Wanfang Database, China National Knowledge Infrastructure (CNKI), without the limitation of publication status and language until September 1, 2020. The systematic review will also search will also search for identify publications, meeting minutes, and grey literature (including unpublished meeting articles). The following search terms are: facial corticosteroid addictive dermatitis, tacrolimus, corticosteroid, glucocorticosteroid, corticosteroid, steroid. The search strategy for PubMed is shown in Table 1.

**Table 1:**
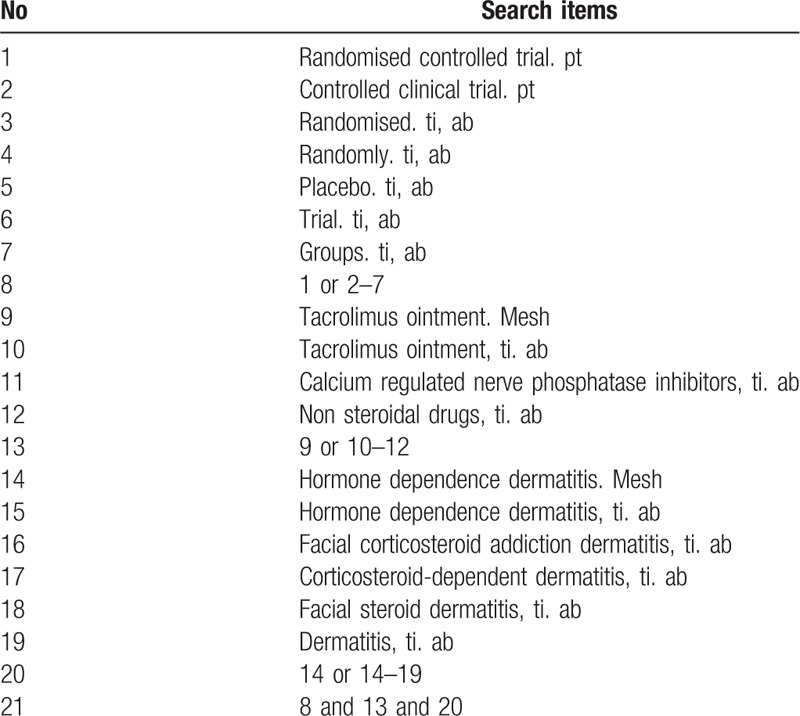
Search strategy used in PubMed.

#### 2.2.4 Data collection and analysis

**Figure 1:**
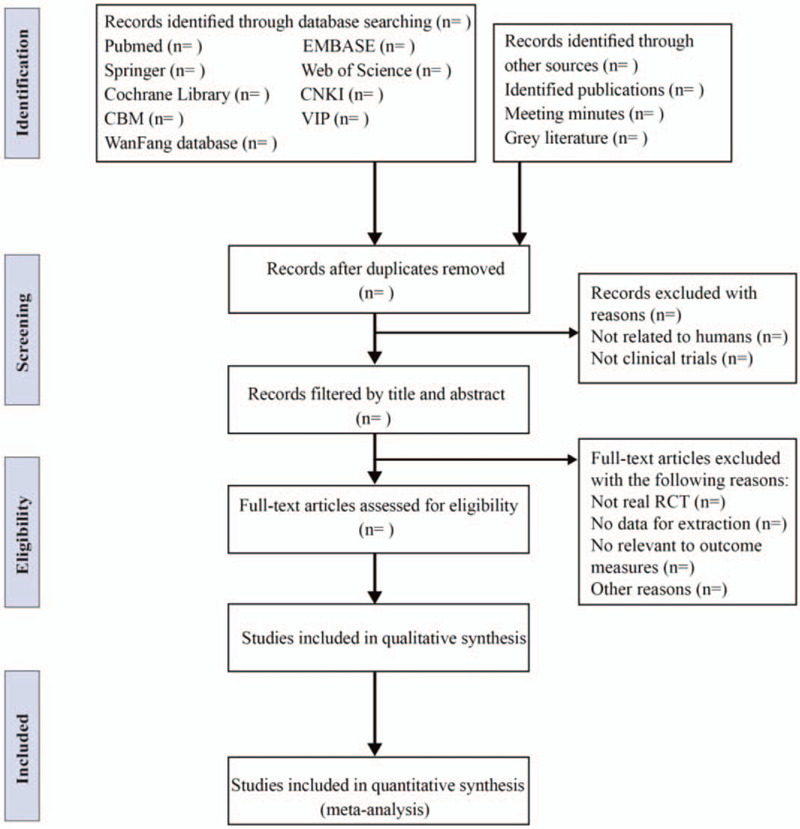
Flow diagram of studies identified.

Before to the literature search, we will organize relevant training to ensure consistency in the evaluation of this study. During the screening of the literature, we will use the EndNote X9 document management software. According to the PRISMA flow chart, 2 researchers (ML and JJD) will strictly follow the inclusion criteria, independently screen all retrieved studies, for the preliminary research selection, only the title and abstract will be reviewed to exclude publications that are clearly inappropriate. If available, the further evaluated by reading the full-text study will be carried, while unqualified studies will be deleted, and the reasons for deletion will be recorded. Each eligible trial will contain the following message: the first author, year of publication. Any disagreement on data relevance will be resolved by the third reviewer. The study flow diagram is shown in Figure 1.

#### 2.2.5 Assessment of risk of bias

Cochrane Collaborations tool will be used to assess RCTs by 2 authors. We will access the evaluation sequence from the following aspects: hiding the allocation method, blindness of participants and staff, number of lost follow-up participants in each group, loss reasons, selective result report and other bias sources, and selective result report. We will use Rev Man 5.3.5 software to generate deviation risk map according to the risk information of deviation assessment included in the study, and discuss the results and impacts critically. For unclear data, we will try to contact the author. The final decisions on any disagreement will be made by a third reviewer through discussion.

#### 2.2.6 Measures of treatment effect

For continuous data, we will use the mean difference (MD) with 95% CIs to calculate the impact size. Also, when binary data is involved, the associated risk (RRs) with 95% CIs will be applicable. Risk ratio and random-effects models will be used if significant heterogeneity is found.

### 2.3 Dealing with missing data

Two reviewers (WT and ML) will contact the authors by e-mail or telephone for the missing data, and we will analyze the available data. If data are unobtainable, the existing data will be analyzed and the potential impact of missing data will be discussed. If necessary, we will conduct a sensitivity analysis to assess the sensitivity of the results to reasonable changes in assumptions.

#### 2.3.1 Assessment of heterogeneity

Clinical heterogeneity will be evaluated by X^2^ and *I*^2^ statistical tests, which conducted in forest areas by using RevMan V.5.3. *P* value *I*^2^ less than 50% will be regarded as homogeneous. On the other hand, *P* value *I*^2^ more than 50% indicates that there is a considerable heterogeneity among studies. At that time, it will be assessed by sensitivity and subgroup analyses.

#### 2.3.2 Assessment of reporting biases

Reporting biases will be accessed by using funnel plots, if more than 10 studies are included in the study.

#### 2.3.3 Data synthesis

The results calculated using Review Manager V5.3 will be analyzed according to the level of statistical heterogeneity: if the *I*^2^ test is less than 50%, the fixed effects model will be used to summarize the data; while the *I*^2^ test is more than 50%, we will choose random-effects model.

#### 2.3.4 Subgroup analysis

If necessary, we will carry out a subgroup analysis based on the age, diagnostic criteria, duration of treatment and interval, or quality of studies, etc.

#### 2.3.5 Sensitivity analysis

Sensitivity analysis will be conducted to assess the robustness of major decisions made during the audit. The sensitivity analysis will focus on research characteristics or types, such as methodological weaknesses and missing data, and more poor quality research or unblinded research will be excluded.

## 3 Discussion

Tacrolimus, as a non-hormone immunosuppressant, can block the activation of T lymphocytes, effectively inhibit the transcription of granulocyte-macrophage colony-stimulating factor (GM-CSF), interleukin-3 and interleukin-4 genes, and then inhibit the release of inflammatory mediators, significantly improve the skin barrier function, thus improving the skin symptoms of facial hormone-dependent dermatitis patients.^[17,18]^ However, with the long-term use of tacrolimus ointment, it has been found that the effect of tacrolimus ointment for facial hormone-dependent dermatitis is slow, and it is easy to relapse after stopping the medication. At present, the efficacy and safety of tacrolimus ointment for facial hormone-dependent dermatitis have not been systematically reviewed, which published in English. It will be of great significance if this review can provide more convincing evidence to help clinicians deal with facial hormone-dependent dermatitis in the decision-making process.

## 4 Footnotes

**State:** We has completed the PRISMA-P checklist^[19]^ when writing our report of the protocol.

### 4.1 Patient and public involvement

No patients nor the public were involved in the development of the research question or study design and will not be involved in recruitment or conduct of the study.

### 4.2 Ethics approval and consent to participate

This systematic review will be based on published data, and thus there is no requirement for ethics approval. The results will be shared through publication in a peer reviewed journal and through presentations at academic conferences.

## Author contributions

**Administrative support:** Ping-Sheng Hao.

**Collection and assembly of data:** Wen Tan, Mao Lei.

**Conception and design:** Mao Li.

**Data analysis and interpretation:** Jingjing Du, Mao Lei.

**Final approval of manuscript:** Ping-Sheng Hao.

**Manuscript writing:** Mao Li, Wen Tan, Jingjing Du, Qiuyue Wang, Linyue Wang, Min Lei, Ping-Sheng Hao.

**Provision of study materials:** Qiuyue Wang, Linyue Wang.
